# Beyond the Bedside: Machine Learning-Guided Length of Stay (LOS) Prediction for Cardiac Patients in Tertiary Care

**DOI:** 10.3390/healthcare12111110

**Published:** 2024-05-29

**Authors:** Sarab AlMuhaideb, Alanoud bin Shawyah, Mohammed F. Alhamid, Arwa Alabbad, Maram Alabbad, Hani Alsergani, Osama Alswailem

**Affiliations:** 1 Department of Computer Science, College of Computer and Information Sciences, King Saud University, P.O. Box 266, Riyadh 11362, Saudi Arabia; 442202754@student.ksu.edu.sa; 2 Healthcare Information Technology Affairs (HITA), King Faisal Specialist Hospital & Research Center, P.O. Box 3354, Riyadh 11211, Saudi Arabia; mfalhamed@moi.gov.sa (M.F.A.); arwa@kfshrc.edu.sa (A.A.); malabbad@kfshrc.edu.sa (M.A.); alswailem@kfshrc.edu.sa (O.A.); 3 Heart Center, King Faisal Specialist Hospital & Research Center, P.O. Box 3354, Riyadh 11211, Saudi Arabia; hani@kfshrc.edu.sa

**Keywords:** cardiac patients, length of stay, machine learning, regression, ensemble learning, sustainability, tertiary care

## Abstract

Efficient management of hospital resources is essential for providing high-quality healthcare while ensuring sustainability. Length of stay (LOS), measuring the duration from admission to discharge, directly impacts patient outcomes and resource utilization. Accurate LOS prediction offers numerous benefits, including reducing re-admissions, ensuring appropriate staffing, and facilitating informed discharge planning. While conventional methods rely on statistical models and clinical expertise, recent advances in machine learning (ML) present promising avenues for enhancing LOS prediction. This research focuses on developing an ML-based LOS prediction model trained on a comprehensive real-world dataset and discussing the important factors towards practical deployment of trained ML models in clinical settings. This research involves the development of a comprehensive adult cardiac patient dataset (SaudiCardioStay (SCS)) from the King Faisal Specialist Hospital & Research Centre (KFSH&RC) hospital in Saudi Arabia, comprising 4930 patient encounters for 3611 unique patients collected from 2019 to 2022 (excluding 2020). A diverse range of classical ML models (i.e., Random Forest (RF), Extreme Gradient Boosting (XGBoost), Light Gradient Boosting Machine (LGBM), artificial neural networks (ANNs), Average Voting Regression (AvgVotReg)) are implemented for the SCS dataset to explore the potential of existing ML models in LOS prediction. In addition, this study introduces a novel approach for LOS prediction by incorporating a dedicated LOS classifier within a sophisticated ensemble methodology (i.e., Two-Level Sequential Cascade Generalization (2LSCG), Three-Level Sequential Cascade Generalization (3LSCG), Parallel Cascade Generalization (PCG)), aiming to enhance prediction accuracy and capture nuanced patterns in healthcare data. The experimental results indicate the best mean absolute error (MAE) of 0.1700 for the 3LSCG model. Relatively comparable performance was observed for the AvgVotReg model, with a MAE of 0.1703. In the end, a detailed analysis of the practical implications, limitations, and recommendations concerning the deployment of ML approaches in actual clinical settings is presented.

## 1. Introduction

Efficient management of resources is vital for hospitals to provide high-quality healthcare while ensuring sustainability [1]. With limited resources, hospitals must effectively allocate them to optimize patient care. One key aspect is the length of stay (LOS), which measures the duration a patient spends in the hospital from admission to discharge [2]. LOS directly impacts patient outcomes, costs, and resource utilization. Prolonged LOS, often seen in complex cardiovascular cases and elderly patients, poses challenges to hospital efficiency and resource management [3,4]. Precisely predicting the LOS in hospitals offers substantial potential to improve efficiency and effectiveness in healthcare settings [5]. Accurate LOS estimation brings various advantages. Firstly, hospitals can diminish unplanned re-admissions by proactively addressing factors prolonging a patient’s stay [6]. Secondly, precise LOS prediction ensures appropriate staffing levels, guaranteeing optimal care provision. Additionally, it aids in informed decisions about discharge plans, promoting smoother transitions and care continuity [7]. Identifying patients at risk of extended stays enables optimized treatment plans and early interventions, curbing complications like hospital-acquired infections [8]. Furthermore, optimizing LOS supports sustainability in healthcare [9] by reducing waste [10] and enhancing overall care quality. In summary, the motivations behind this research are rooted in the critical need for efficient management of hospital resources to ensure high-quality healthcare delivery and sustainability. With hospitals facing constraints in resource allocation, accurate prediction of LOS emerges as a crucial factor directly impacting patient outcomes and resource utilization. Prolonged LOS not only poses challenges to hospital efficiency but also affects patient care and costs. By accurately predicting LOS, hospitals can mitigate re-admissions, optimize staffing levels, and facilitate informed discharge planning, ultimately improving patient care and operational efficiency.

Conventional methods for LOS prediction in hospitals have traditionally relied on statistical models and clinical expertise. These methods often involve statistical analysis, where historical patient data, including demographics, medical history, diagnosis, and treatment details, are utilized to estimate the LOS. Additionally, scoring systems like the Charlson Comorbidity Index [11] or the Acute Physiology and Chronic Health Evaluation (APACHE) [12] score are commonly employed to assess patient severity and predict LOS based on disease complexity and comorbidities. Clinical pathways and protocols are also utilized to estimate LOS for specific conditions or procedures, guiding the expected course of patient care and discharge planning [13]. While these conventional approaches provide a foundational framework for LOS prediction, they often lack the granularity and adaptability required to accurately forecast LOS for individual patients in dynamic healthcare environments. Recent advancements in machine learning (ML) technologies offer promising avenues for enhancing LOS prediction in hospitals. ML algorithms can effectively analyze vast amounts of patient data to discern complex patterns and relationships that may influence LOS. Furthermore, ML algorithms can adapt and improve over time by continuously learning from new data, thereby enhancing prediction accuracy and reliability. By leveraging big data analytics and predictive modeling, healthcare systems can develop proactive interventions and personalized care plans tailored to individual patient needs, thereby mitigating factors contributing to prolonged LOS and improving overall efficiency and quality of care.

In the context of LOS prediction, the existing literature employed ML models including Support Vector Machine (SVM), Decision Tree (DT), artificial neural network (ANN), Bayesian Network, Convolutional Neural Network (CNN) and ensemble learning [14,15,16,17] for diverse range of datasets (i.e., King Abdul Aziz Cardiac dataset [4], Microsoft dataset [18], New York Hospitals dataset [19], United States Hospital dataset [20], Royal Adelaide Hospital dataset [21]), demonstrating significant potential of ML techniques towards addressing the problem. However, most of the existing literature treats LOS prediction as a classification problem, overlooking the nuanced details of LOS duration. Moreover, a significant gap exists in incorporating patients’ medical histories, stored at transaction levels in electronic medical records, into predictive models. This oversight hampers the comprehensive understanding of patients’ health trajectories and may lead to sustainability challenges in healthcare systems. Additionally, prevailing studies typically fail to capitalize on the predictive capabilities inherent in the temporal order of medical events within hospital encounters, thereby limiting the accuracy and reliability of LOS predictions. There are dataset limitations in terms of the number of relevant features, quality, and size, which hinders the real-world deployment of ML models in actual clinical settings.

Emphasizing the real-world practical deployment of ML techniques for LOS prediction, this study aims to develop a comprehensive real-world dataset of adult cardiac patients in tertiary care settings and implement multiple ML techniques inspired by the literature (i.e., Random Forest (RF) [22], Extreme Gradient Boosting (XGBoost) [23], Light Gradient Boosting Machine (LGBM), ANNs [24], Average Voting Regression (AvgVotReg), Two-Level Sequential Cascade Generalization (2LSCG), Three-Level Sequential Cascade Generalization (3LSCG), and Parallel Cascade Generalization (PCG)) as a tool to predict the LOS. This study further contributes the practical considerations, implications, and recommendations towards the actual deployment of ML techniques for LOS prediction in real-world clinical settings. The methodology encompasses various dimensions of data availability, including information at admission, during treatment, and preceding discharge orders. Utilizing real-world data from cardiac patients at the King Faisal Specialist Hospital & Research Centre (KFSH&RC) in Saudi Arabia, referred to as the SaudiCardioStay (SCS) dataset, the dataset comprises patient demographics, clinical features, and treatment records. KFSH&RC, a leading healthcare institution in the Middle East, boasts multiple branches across Riyadh, Jeddah, and Madinah, with over 2200 beds and a team of 15,000 staff and clinical consultants. The dataset comprises 16 subsets detailing demographic and diagnostic information from patient encounters spanning 2019, 2021, and 2022. Excluding 2020 due to the COVID-19 pandemic’s significant impact, resulting in limited admissions, especially for critical cases, the study focuses on recent years to capture evolving treatment plans and equipment usage potentially affecting LOS. The dataset encompasses 4930 patient encounters for 3611 unique patients, each encounter representing a distinct visit. Diverging from prior studies overlooking the temporal sequence of hospital events, this research leverages the predictive potential of patients’ medical histories. By considering the chronological order of medical events, this study aims to tap into the latent predictive insights offered by patients’ medical backgrounds. The anticipated contributions of the manuscript are as follows:

- Development of comprehensive adult cardiac patient dataset (i.e., SaudiCardioStay (SCS)) from KFSH&RC hospital in Saudi Arabia consisting of 4930 patient encounters for 3611 unique patients collected from 2019–2022 (excluding 2020).
- Implementation of a diverse range of classical ML techniques including RF, XGBoost, LGBM, ANNs, and AvgVotReg for LOS prediction using the SCS dataset.
- Introduction of a novel approach for LOS prediction by incorporating a dedicated LOS classifier within a sophisticated ensemble methodology (i.e., 2LSCG, 3LSCG, PCG). By fusing insights from both classification and regression domains, the research aims to enhance prediction accuracy and capture nuanced patterns in healthcare data, ultimately improving clinical decision support and patient care management.
- Detailed analysis of the practical implications, limitations, and recommendations in the context of the practical deployment of ML approaches in actual clinical settings.

## 2. Related Work

Accurate knowledge of patient discharge dates upon admission is an essential tool for efficient patient care. To this end, numerous past studies have concentrated on devising solutions for predicting in-hospital LOS. However, previous research efforts aimed at predicting LOS have seldom been tailored to a specific patient cohort, such as those with cardiac disease [1,4,6,25]. This section presents a review of notable studies in the context of LOS prediction using ML to demonstrate the state of the art in this domain. The review is structured in chronological order to better understand the advancements over time.

In terms of LOS prediction for cardiac patients, in 2007, Rowan et al. [6] proposed an ensemble learning approach combining multiple ANNs to classify cardiac patients into various risk categories based on their in-hospital LOS. In 2013, Hachesu et al. [25] employed ensemble learning to classify the LOS of cardiac patients by combining three machine learning algorithms: DT, ANN, and SVM. The data used were limited to demographic and clinical data. Later in 2016, Tsai et al. [1] employed linear regression and ANNs to predict the in-hospital LOS for patients with cardiac diseases. Notably, temporal data were not considered in their study. In 2019, Daghistani et al. [4] also explored the application of ML techniques for classifying the LOS of patients with cardiac diseases into short, intermediate, or long categories. Specifically, the authors employed RF, ANNs, SVM, and Bayesian network models on a dataset obtained from the King Abdulaziz Cardiac Center at the King Abdulaziz Medical City Complex in Riyadh, Saudi Arabia. The RF model exhibited superior performance compared to the other models, with an accuracy of 0.80%. In one of the most recent works of research, Abdurrab et al. [17] evaluated Bayesian regression models and ML regression models for predicting the LOS of cardiac patients undergoing cardiac bypass surgery at Tabba Heart Institute, Karachi, Pakistan, between 2015 and 2020. Hierarchical Bayesian regression outperforms simple Bayesian regression and ML models in accuracy (RMSE = 1.49, MAE = 1.16) and interpretability, particularly in handling data variability and extreme values without removing outliers. It emphasizes the importance of accurate LOS prediction for operational efficiency in medical institutions, offering risk stratification capabilities for clinical decision making based on high-risk features affecting LOS. Hierarchical Bayesian models are praised for their ability to handle heterogeneous, continuous variables and assess variable effects across different LOS ranges, facilitating resource allocation and improved patient care.

On the other hand, several researchers have focused on general datasets considering multiple diseases for the prediction of in-hospital LOS. In 2016, Barnes et al. [7] employed logistic regression and RF models to predict the likelihood of a patient being discharged by 2 p.m. or the end of the day, respectively. In 2017, Turgeman et al. [26] proposed a Cubist linear regression model for predicting in-hospital LOS. Their investigation focused on determining the feasibility of using SVMs to distinguish cases with higher Cubist error from those with lower Cubist error. In the same year, Lior et al. [26] explored the application of a regression tree model called Cubist for predicting LOS at the time of admission using static inputs available at admission, without changes during the patient’s hospitalization. Trained and validated on de-identified administrative data from Veterans Health Administration (VHA) hospitals in Pittsburgh, PA, the Cubist model outperformed alternative techniques in accuracy. This model partitions the dataset and estimates linear regressions for each partition, allowing for a deeper understanding of factors correlated with LOS. Results suggest higher prediction errors for patients with more frequent recent admissions or longer previous hospital stays. Additionally, a Radial Basis Function (RBF) kernel was used to map cases into a higher dimensional space, facilitating the separation of cases based on their level of Cubist error using an SVM algorithm. The interpretability of Cubist predictions provides insight into the classification rules applied to LOS data and underlying factors influencing LOS, offering valuable information for early interventions and resource allocation. In 2018, Livieris et al. [27] developed a decision support system to predict the LOS in hospitals. The system took into account demographic, clinical, and geographical factors and employed a two-stage classifier method. The proposed method included classifiers A and B, with A-level classification into three categories of LOS and B-level classification into sub-categories. The proposed method achieved 78.53% accuracy. In 2019, Steele and Thompson [28] proposed a methodology for classifying in-hospital LOS into two categories: less than eight days and more than or equal to eight days. They employed various traditional ML algorithms and ensemble approaches. Bayesian networks outperformed all other algorithms.

In 2020, Mekhaldi et al. [18] utilized RF and gradient boosting algorithms to predict in-hospital LOS using an open-source Microsoft dataset. Siddiqa et al. [19] utilized six regression algorithms with data from New York Hospitals, comprising information from over 2.3 million patients with different diseases. The RF outperformed other techniques. The data did not include temporal information as well. In the same year, Bacchi et al. [21] employed CNN [29,30] and ANNs to classify in-hospital LOS into two categories: patients staying less than or equal to two days and those staying more than two days. They utilized a dataset comprising doctors’ notes from the Acute Medical Unit of a tertiary hospital, The Royal Adelaide Hospital, for a two-month period in 2019. The ANN outperformed the CNN model in the LOS classification task. In 2021, Triana et al. [31] employed traditional ML algorithms to predict the LOS for coronary artery bypass grafting patients after cardiac surgery. Several other researchers have proposed ML models for predicting the LOS of general-category intensive care unit (ICU) and post-surgical patients [32,33,34,35,36]. In 2022, Zolbanin et al. [20] developed a deep neural network model utilizing a multi-layer perceptron with different hidden layers to predict in-hospital LOS for pneumonia and chronic obstructive pulmonary disease patients. They utilized a vast dataset encompassing patient encounters from over 180 hospitals across the United States, spanning a five-year period from 2010 to 2015. The dataset comprised clinical data extracted from medical images, physician notes, laboratory results, and administrative data. Notably, the assessment criteria for the LOS predictions included a tolerance range of ±2 days and ±3 days to account for prediction errors. The proposed method is exhaustive and uncommon in the ML literature related to the prediction of LOS. In 2023, Al-Tawil et al. [37] examined the application of bio-inspired metaheuristic algorithms for predicting type 2 diabetes using medical datasets. The focus was on comparing the performance of the Cuttlefish Algorithm (CFA) and the genetic algorithm in feature selection and their integration with various classifiers. By leveraging two prominent datasets, the Pima Indian Diabetes (PID) dataset and the Hospital Frankfurt Diabetes (HFD) dataset, the study demonstrated the superior accuracy of the CFA, particularly with larger datasets. The CFA’s efficacy in optimizing feature selection stems from its inspiration from the cuttlefish’s color-changing behavior, leading to improved classification results. The research highlighted the importance of efficient feature selection in handling massive, complex medical datasets to enhance prediction accuracy and computational efficiency. In a recent work of research, Jaotombo et al. [38] investigated the utility of ML models in predicting prolonged hospital length of stay (PLOS) using data from the French Medico-Administrative database (PMSI). Through a retrospective cohort study of discharges from a large university hospital in France in 2015, diverse ML algorithms such as logistic regression (LR), classification and regression trees (CARTs), RF, gradient boosting (GB), and neural networks (NNs) were employed to forecast PLOS based on LOS thresholds. Results demonstrated that the GB classifier achieved the highest performance, outperforming other models significantly. Key predictors of PLOS included the patient’s post-hospitalization destination, highlighting the importance of targeted resource allocation. This study extends the existing literature by showcasing the effectiveness of ML, particularly the GB algorithm, in PLOS prediction, offering valuable insights for healthcare planning and resource management.

Generative AI has recently emerged as a disruptive technology revolutionizing many application domains [39,40,41]. In 2022, Mantas et al. [40] reassessed the Russian GPT-3 (ruGPT-3) model for predicting the LOS in neurosurgery using narrative medical records, comparing its performance to doctors’ and patients’ expectations. Doctors had the most accurate LOS predictions (MAE = 2.54), while ruGPT-3’s predictions (MAE = 3.53) were close to patients’ expectations (MAE = 3.47) but statistically inferior (*p* = 0.011). Despite the solid performance of ruGPT-3, there is room for improvement compared to previous models using recurrent neural networks and FastText vector representations. Future research should integrate unstructured textual data with structured features to further enhance model accuracy. In 2023, Peng et al. [41] explored the application of Large Language Models (LLMs) in healthcare by developing GatorTronGPT, a generative clinical LLM trained on 277 billion words of text. This included 82 billion words of clinical text from the University of Florida Health and 195 billion words of diverse general English text. Using a GPT-3 architecture with up to 20 billion parameters, GatorTronGPT was designed for biomedical natural language processing (NLP) and healthcare text generation. Results demonstrated that GatorTronGPT significantly enhanced biomedical NLP capabilities. Notably, synthetic NLP models trained on text generated by GatorTronGPT outperformed those trained on real-world clinical text.

Most of the existing literature on LOS prediction in hospital settings predominantly treats it as a classification problem [4,6,7,21,25,28,32,33,38]. Choosing between continuous and categorical LOS prediction methods can significantly impact hospital operations. While continuous prediction offers a detailed view of LOS by estimating precise durations, its implications for hospital sustainability vary depending on context. Healthcare sustainability involves complex factors like resource allocation and patient care. Incorporating temporal data, particularly patients’ medical histories, is crucial for accurate LOS prediction. However, there is a noticeable gap in integrating these histories into predictive models despite electronic medical records capturing such data at transactional levels [20]. Neglecting patients’ medical histories could pose sustainability challenges for healthcare systems. Temporal data, including past admissions, LOS, and time since previous surgeries, offer valuable insights into patients’ health trajectories and potential LOS.

This study adopts a unique approach by prioritizing precise duration estimation for hospital stays over categorical classification. Unlike previous studies that often overlook the temporal order of events in hospital encounters, this research capitalizes on the predictive potential inherent in patients’ medical histories. By acknowledging and incorporating the temporal aspect of medical events, this study aims to unlock the untapped predictive insights offered by patients’ medical histories.

## 3. Dataset Description and Preparation

The dataset used for this work was constructed from admission data at KFSH&RC for the years 2019, 2021, and 2022, excluding the year 2020 due to the influence of COVID-19. Various features were included based on best practices, such as demographics, health conditions (e.g., heart failure and diabetes), prior surgeries, prior admissions, previous diagnoses, and the latest blood tests. Table 1 shows the full initial feature set, including a description of each feature, the number of distinct values for categorical features and the range for numerical features (Domain), the percentage of missing values (PMV), and the feature type (categorical, numerical, or date/time). The initial dataset included 18,869 encounters for 8002 unique patients. The decision to use a dataset from a single hospital in this study was driven by several key factors. Using KFSH&RC data ensures relevance and specificity to local patient population, treatment protocols, and operational processes, enhancing the model’s accuracy in predicting LOS. It also guarantees compliance with data privacy regulations, which restrict the use of external patient data. Furthermore, data are collected using consistent methods and standards, ensuring high quality and reliability, whereas external datasets may introduce variability and inconsistencies. Lastly, data collected from a single institute facilitate seamless integration and immediate benefits within a hospital’s operational workflow. While national and international datasets exist, their use is limited by privacy concerns, potential inconsistencies, and a lack of direct applicability to specific contexts.

It is important to note that while collecting a large amount of data is beneficial for training machine learning models, including excessive data can introduce noise and lead to predictions that mirror the long, unnecessary hospital stays. This can hinder the model’s ability to accurately assess the actual duration of stays. Therefore, a careful selection of relevant features and data points was made to ensure the model’s effectiveness in predicting hospital stays while avoiding unnecessary complexity and bias.

Several features were constructed for patient admission and surgery, including features related to previous admissions, surgery-related features, and features that report whether vital sign readings were within the normal range. The features related to previous admissions include HAS-PRIOR-ADMISSION, which indicates whether a patient had previous admissions, and PRIOR-LOS-DAYS, which reports the length of stay associated with the previous admission. LAST-YEAR-ADMISSION-COUNT reports the number of admissions in the past year, and LAST-ADMISSION-DAYS reports the number of days since the past admission. Similarly, the surgery-related features include HAS-PRIOR-SURGERY, HAS-PRIOR-SURGERY-60-DAYS, and LAST-SURGERY-DAYS. The features that report whether vital sign readings were within the normal range are based on the Cleveland Clinic’s standards for normal vital signs [42]. The count features include the TASK-ASSAY-COUNT feature, which denotes the number of lab tests performed for each patient, and LAST-MONTH-TASK-ASSAY-COUNT, which signifies the number of laboratory tests administered within the previous month. AGE was calculated by subtracting BIRTH-DT-TM from ARRIVE-DT-TM.

Several filters were employed to refine the dataset. The NURSE-UNIT feature was filtered to only include records from cardiac units. The ENCOUNTER-TYPE and DISCHARGE-DISPOSITION features were filtered to only include encounters categorized as ‘inpatient’ and ‘Alive and discharge with approval’, respectively. The DISCHARGE-TO-LOCATION feature was filtered to only include records with the ‘Home’ category. Encounters from 2018 and 2020 were excluded from the training data, but the associated data were used for constructing features such as HAS-PRIOR-ADMISSION. Only patients aged 14 years or older were included.

The dataset contained missing data for three features, RESULT_VALUE_NUMERICAL, PRIOR_ADMISSION_ARRIVE, and PRIOR_ADMISSION_DISCHARGE (Table 1). To deal with missing values in RESULT_VALUE_NUMERICAL, the filtering process was applied to the TASK-ASSAY feature, focusing on selecting crucial blood tests essential for clinical assessment. Notable tests that retained post-filtration include Hgb, WBC, Creat, e-GFR, Hgb A1c percent, Na, Pro-Brian Natriuretic Peptide, and Troponin-T. To rectify the issue of null values within the ’PRIOR_ADMISSION_DISCHARGE’ and ’PRIOR_ADMISSION_ARRIVE’ features, a systematic recalibration process was devised. This involved the creation of two new features, namely ’PRIOR_ARRIVE_DT_TM’ and ’PRIOR_DISCH_DT_TM’, wherein null values were replaced based on preceding values within the dataset. Initially, a method named ’Add_Prior_Admission_date’ was developed to facilitate this recalibration. This method generated a new dataframe, ’PRIOR_ARRIVE_DT_TM’, wherein each value was shifted by one cell and forward-filled to replace null entries. Likewise, ’PRIOR_DISCH_DT_TM’ was created using a similar approach. Subsequently, these newly generated features were integrated into the ’Encntr_df’ dataframe, thereby incorporating them into the dataset. Following this integration, a merge operation was executed between ’Encntr_df’ and ’prior_admission_df’ to further refine the dataset. Despite the recalibration efforts, residual null values may persist within ’PRIOR_ARRIVE_DT_TM’ and ’PRIOR_DISCH_DT_TM’ in ’Encntr_df’. In such instances, if the corresponding ’ECNTR_ID’ in ’prior_Admission_df’ contains non-null values, these values are utilized to replace null entries in the merged dataset. Outliers in target features were dealt with by combining the interquartile range (IQR) method with boxplots. Boxplots were used to detect outliers in the above-mentioned features. The IQR method was then employed to identify outliers falling outside the upper and lower bounds. Values less than the lower bound were replaced with the 10th percentile value, and values greater than the upper bound were replaced with the 90th percentile value. Similarly, the IQR method was applied to handle outliers that fell within the same interval of (10%, 90%).

**Figure 1:**
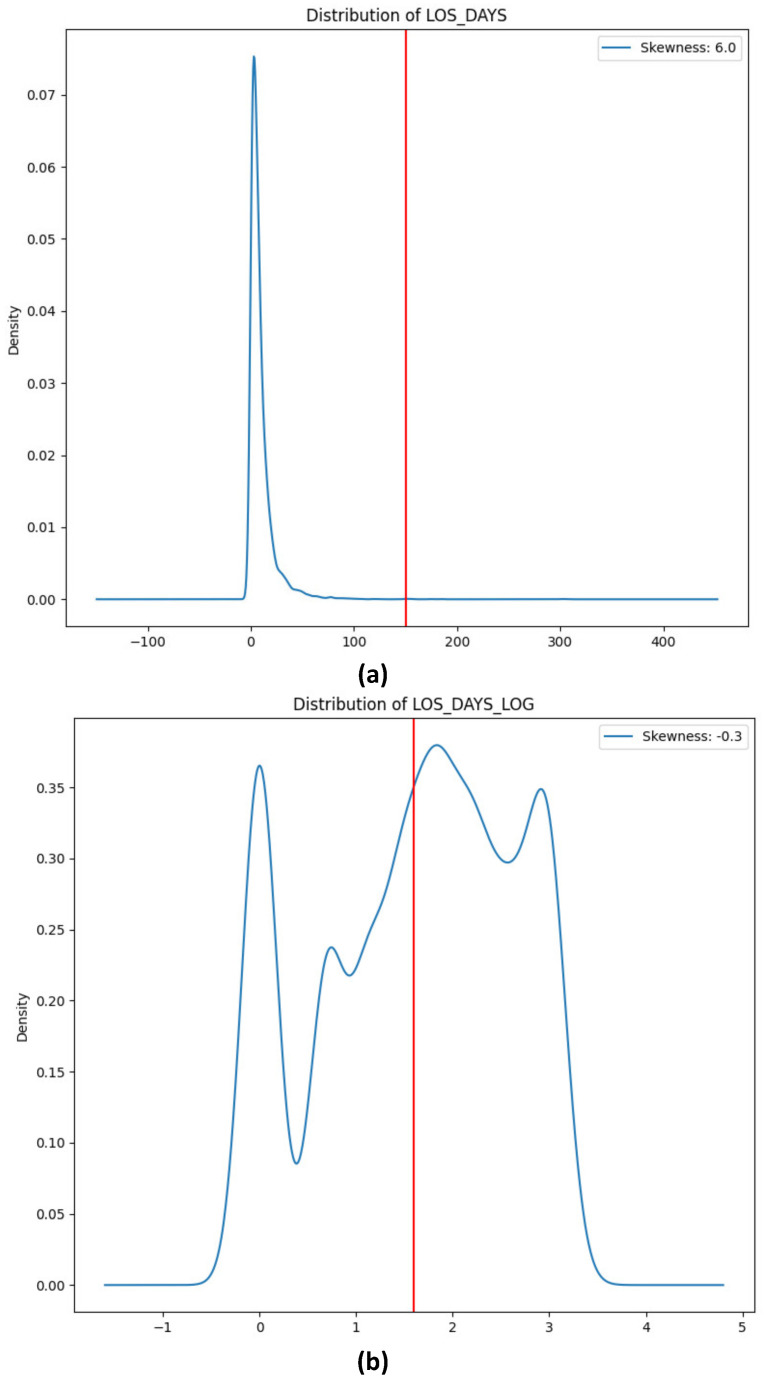
The distribution and skewness of LOS-DAYS. (**a**) Before preprocessing. (**b**) After log transformation.

The distribution of LOS-DAYS is shown in Figure 1, with the red vertical line highlighting the mean of the distribution. The distribution was skewed with a right tail (Figure 1a). To address this issue, a log transformation was applied to transform the skewed distribution into a normal distribution. Figure 1 depicts the distribution of LOS-DAYS after log transformation (Figure 1b). This was performed for all features related to LOS-DAYS, such as LAST-YEAR-LOS-MAX and PRIOR-LOS-DAYS.

**Figure 2:**
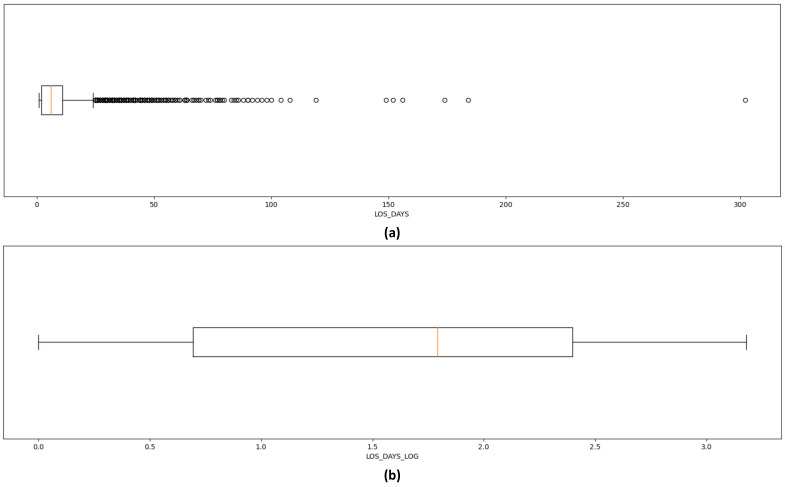
Outlier detection of the target feature LOS-DAYS: (**a**) boxplot of the original feature; (**b**) boxplot of the feature after log transformation and handling of outliers.

Figure 2 depicts the boxplots of LOS-DAYS, both before (left) and after (right) outlier handling. In regards to other noises than outliers, in the collection of datasets, an automated process was adopted to capture the readings (e.g., temperature, blood pressure, heart rate) from the devices. Further to this, other variables such as admission mode, admission type, and nurse unit were selected from a drop-down menu, hence significantly reducing the likelihood of noise.

The SOURCE-CATEGORY feature was categorized into four groups based on the clinical decision of Cardiac Consultants at KFSH&RC. Numerical features were normalized using the Min-max normalization method. Dummy encoding was performed for all multi-categorical features. Null values in LAST-YEAR-ADMISSION-COUNT, LAST-SURGERY-DAYS, and  LAST-ADMISSION-DAYS were replaced by zeroes. The learning algorithms that cannot handle missing values in the blood test and vital signs were replaced by ‘−1’ for them.

To utilize the random forest for classification, the target feature of LOS-DAYS-LOG was transformed into a categorical variable. This was performed by creating a new feature called LOS-DAYS-CATEGORY and applying Quantile-Cut (Qcut) to bin the LOS-DAYS-LOG feature into intervals with approximately equal frequency in each bin. The goal was to obtain a balanced number of elements in each bin. The resulting intervals and their corresponding class labels are shown in Table 2. The number of intervals was set to 4. Following the feature engineering steps, the dataset comprised 69 features, representing 3611 unique patients and 4930 encounters.

## 4. Feature Subset Selection

The dataset comprised both categorical and numerical features, and our research involved classification and regression tasks. Therefore, several feature subset selection methods were employed. For instance, Kruskal–Wallis was used to test the correlation between the numerical features and a categorical target variable, while the chi-square test was employed to test the correlation between the categorical features and a categorical target variable for the classification task. On the other hand, the Pearson correlation method was utilized to test the correlation between the numerical features and a numerical target variable, while Kruskal–Wallis was used to test the correlation between the categorical features and a numeric target variable for the regression task.

For the regression task, where LOS is a continuous value, features with a correlation coefficient greater than or equal to 0.1 using the Pearson correlation method were included [43], yielding 18 features as a result. Features with a *p*-value less than or equal to 0.05 in the Kruskal–Wallis test were chosen for further training of the model, yielding 37 features.

For the classification task, where LOS is a categorical variable, features with a *p*-value less than or equal to 0.05 in both the chi-square test and Kruskal–Wallis were selected for further training of the model, resulting in 39 and 24 features, respectively.

Table 3 lists the abbreviations assigned for the different feature subsets (FS-N) used in each trial, along with the feature subset selection method. Categorical targets are indicated by (C), while numerical targets are indicated by (R). In the FS-1 subset, all features are used.

## 5. Research Methodology

**Figure 3:**
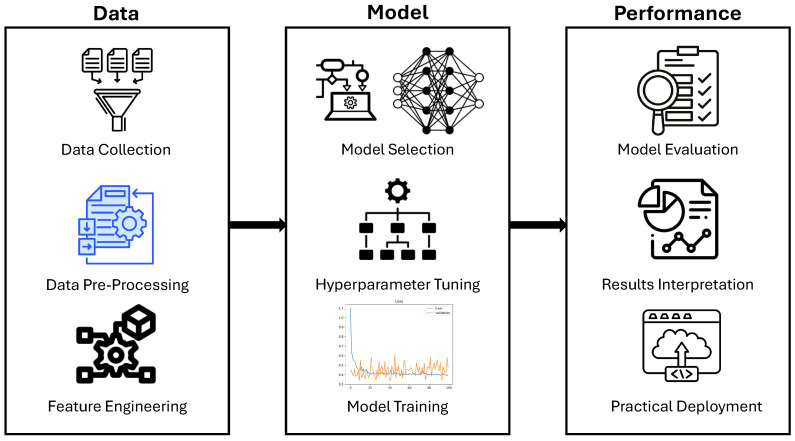
The functional flow diagram for the proposed approach.

The proposed research approach (see Figure 3) begins with the collection of a comprehensive dataset, termed SCS, sourced from KFSH&RC. This dataset spans the years 2019 to 2022, excluding 2020 due to the COVID-19 pandemic’s impact, and encompasses detailed information on adult cardiac patients’ demographics, clinical features, and treatment records. Subsequently, preprocessing steps are applied to ensure data quality and compatibility for machine learning models, including handling missing data through imputation methods, encoding categorical variables, and normalizing numerical features. Relevant features are then extracted from the dataset, considering various dimensions of data availability such as admission, treatment, and discharge orders, while incorporating the temporal sequence of medical events to capture predictive insights from patients’ medical histories.

Following feature engineering, multiple machine learning techniques are implemented, including RF, XGBoost, LGBM, ANNs, AvgVotReg, and ensemble methods like 2LSCG, 3LSCG, and PCG. These models are trained on the SCS dataset to predict LOS, with performance evaluation conducted using metrics such as root mean square error (RMSE) and mean absolute error (MAE) to enable comparison and selection of the most effective approach. In training very deep multi-layer neural networks, a common challenge known as vanishing gradients can arise. This phenomenon occurs when the gradients of the loss function with respect to the parameters of the network become extremely small as they propagate backward through the layers during the training process. As a result, the weights of early layers receive minimal updates, hindering the convergence of the network and impeding its ability to learn effectively. To mitigate the issue of vanishing gradients, a specialized algorithm known as stochastic loss descent is introduced into the training process. This algorithm dynamically adjusts the learning rate based on the orientation of the gradient vector, allowing for more substantial updates of weights in very deep networks. By adapting the learning rate proportionally to the gradient direction, the algorithm ensures that updates are more significant in directions where gradients are large, thus preventing the gradients from vanishing and facilitating more effective training of deep neural networks. A detailed analysis of practical implications, limitations, and recommendations for deploying machine learning techniques in real-world clinical settings is undertaken, encompassing considerations such as data privacy regulations, model interpretability, and integration with existing healthcare workflows.

## 6. Background to Machine Learning Architectures

For this study, we explored seven distinct regression architectures, including three regressors: RF, XGBoost, and a two-layer ANN. We additionally investigated four ensemble architectures: AvgVotReg, 2LSCG, 3LSCG, and PCG. In all of the cascade generalization architectures, an RF classifier was utilized as the classifier in the first level, while any of the following learners were employed in the regressor modules: ANN, RF, and XGBoost.

### 6.1. Random Forest (RF)

RF [22] is an ensemble learning technique that combines the power of multiple decision trees to make predictions. Each decision tree is built on a randomly sampled subset of the training data, and the final prediction is obtained by aggregating the predictions of all individual trees. This aggregation can be performed through averaging (for regression tasks) or voting (for classification tasks).

The foundation of RF lies in the bagging (bootstrap aggregating) principle, which involves creating multiple bootstrap samples (sampling with replacement) from the original dataset. Each bootstrap sample is used to train a separate decision tree. By allowing each tree to be trained on a different subset of the data, RF promotes diversity among the trees, which helps to reduce the variance of the overall model and improve generalization performance.

Let us denote our dataset as $D=\{\left(x_{1},y_{1}\right),\left(x_{2},y_{2}\right),\ldots ,\left(x_{n},y_{n}\right)\}$, where $x_{i}$ represents the features of the *i*-th instance and $y_{i}$ represents its corresponding label. In RF, to build each decision tree:

- Random sampling: Randomly select a subset of the dataset with replacement to create a bootstrap sample $D_{b}$. Typically, the size of $D_{b}$ is equal to the size of the original dataset, but some instances may be repeated, while others may be left out.
- Tree construction: Build a decision tree using the bootstrap sample $D_{b}$. At each node of the tree, a random subset of features is considered for splitting. This randomness adds further diversity to the trees and prevents them from being overly correlated.
- Ensemble aggregation: Once all decision trees are constructed, predictions are made for new instances by aggregating the predictions of all trees. For regression tasks, this aggregation is usually performed by averaging the predictions of individual trees. Mathematically, the prediction $\hat{y}$ for a new instance *x* can be expressed as:
  $$\begin{array}{c} \hat{y}=\frac{1}{T}\sum_{1}^{T}f_{i}\left(x\right) \end{array}$$
  where *T* is the total number of trees in the forest and $f_{i}\left(x\right)$ is the prediction of the *i*-th tree.

RF has several hyperparameters that can be tuned to optimize its performance, such as the number of trees in the forest, the maximum depth of each tree, and the number of features considered at each split. Through careful tuning and cross-validation, RF can provide robust and accurate predictions for various machine learning tasks, including both classification and regression.

### 6.2. Extreme Gradient Boosting (XGBoost)

XGBoost is a sophisticated ensemble learning technique renowned for its effectiveness in various machine learning tasks. It belongs to the class of gradient boosting decision tree algorithms, which iteratively improves the performance of an ensemble of weak learners, typically decision trees, by optimizing a predefined loss function. Unlike traditional gradient boosting methods, XGBoost incorporates several advanced features and optimizations, making it a popular choice in both academia and industry. At the core of XGBoost is a greedy algorithmic strategy that constructs decision trees in a level-wise manner. This approach involves recursively partitioning the input space into regions, aiming to minimize the loss function at each step. Moreover, XGBoost employs Taylor’s expansion to approximate the loss function, enabling efficient optimization of the model parameters.

One distinctive aspect of XGBoost is its regularization strategy, which distinguishes it from the original gradient boosting algorithm proposed by Friedman [44]. XGBoost introduces penalties on the complexity of individual trees, discouraging the model from creating overly complex trees that may lead to overfitting. This regularization term is incorporated into the objective function, and its magnitude increases as the complexity of the tree grows. By penalizing complexity, XGBoost encourages the model to prioritize simpler and more interpretable trees, ultimately enhancing the model’s generalization performance on unseen data.

The key components of XGBoost:

- Greedy tree construction: XGBoost builds decision trees in a sequential manner, with each tree attempting to correct the errors made by the previous trees. At each step, the algorithm selects the best split for each node based on the reduction in the loss function. This greedy approach ensures that the model continuously improves its predictive accuracy with each additional tree.
- Regularization: XGBoost incorporates regularization techniques such as L1 (Lasso) and L2 (Ridge) regularization to penalize the complexity of the trees. By adding these penalties to the objective function, XGBoost discourages the growth of overly complex trees, thereby mitigating the risk of overfitting. The regularization term controls the trade-off between model complexity and accuracy, allowing the algorithm to achieve better generalization performance.
- Optimized loss optimization: XGBoost utilizes efficient algorithms for gradient computation and optimization, resulting in faster convergence and reduced training time. By optimizing the loss function using second-order Taylor expansions, XGBoost achieves superior performance compared to traditional gradient boosting methods.

XGBoost is a powerful ensemble learning technique that combines the strengths of gradient boosting with advanced regularization and optimization strategies. Its ability to construct interpretable yet highly accurate models makes it a popular choice for a wide range of machine learning tasks, including classification, regression, and ranking.

### 6.3. Artificial Neural Networks (ANNs)

ANNs [24] represent a class of machine learning models inspired by the biological neural networks found in the brain. ANNs consist of interconnected nodes, or neurons, organized into layers. Each neuron receives input signals, processes them using an activation function, and then transmits the output to neurons in the subsequent layer. In the context of this work, ANNs were employed as a computational tool for learning and predicting complex relationships within the dataset. The architecture of the ANN involved an input layer, one or more hidden layers, and an output layer. The input layer received the features of the dataset, with each neuron representing a specific feature. The hidden layers performed nonlinear transformations on the input data to extract relevant features and capture intricate patterns present in the data. Finally, the output layer produced the network’s predictions based on the processed information from the hidden layers. Training an ANN entails adjusting the weights of the connections between neurons to minimize the error between the predicted output and the actual output (target). This process involves an optimization algorithm, such as stochastic gradient descent, which iteratively updates the weights based on the calculated gradients of the loss function.

The choice of the number of neurons in each hidden layer is critical and often determined through experimentation and hyperparameter tuning. Increasing the number of neurons allows the network to learn more intricate representations of the data but may also lead to overfitting if not regularized properly. In the specific configuration described in this work, the ANN architecture included an input layer with 77 neurons, reflecting the dimensionality of the input data. Additionally, the network comprised two hidden layers with 5 and 70 neurons, respectively. These hidden layers enabled the network to learn complex representations of the data, capturing both low-level and high-level features.

Mathematically, the activation $a_{j}^{l}$ of neuron *j* in layer *l* is computed as a nonlinear function of the weighted sum of inputs:

$$\begin{array}{c} a_{j}^{l}=f\left(\sum_{k}w_{jk}^{l}a_{k}^{l-1}+b_{j}^{l}\right) \end{array}$$

Here, $w_{jk}^{l}$ represents the weight of the connection between neuron *k* in layer l−1 and neuron *j* in layer $l,a_{k}^{l-1}$ is the activation of neuron *k* in the previous layer, $b_{j}^{l}$ is the bias term, and *f* is the activation function.

ANNs offer a versatile framework for various machine learning tasks, including classification, regression, and pattern recognition. By leveraging the principles of distributed representation and hierarchical abstraction, ANNs can effectively model complex relationships in data and make accurate predictions across diverse domains.

### 6.4. Average Voting Regression (AvgVotReg)

The architecture of AvgVotReg is designed to leverage the strengths of multiple regression models—RF, XGBoost, and ANN—to generate robust predictions for the regression task. Firstly, each base learner—RF, XGBoost, and ANN—is trained independently on the regression dataset. During training, these algorithms learn to map the input features to the target variable, capturing different aspects and patterns present in the data.

Let us denote the predictions of the individual base learners as $y_{\mathrm{RF}}$, $y_{\mathrm{XGBoost}}$, and $y_{\mathrm{ANN}}$, respectively, where *y* represents the predicted target variable. After training, the final prediction for the regression task is obtained by averaging the predictions of all base learners:

$$\begin{array}{c} \mathrm{Final}\;\mathrm{Prediction}=\frac{y_{\mathrm{RF}}+y_{\mathrm{XGBoost}}+y_{\mathrm{ANN}}}{3} \end{array}$$

This averaging process combines the predictions from each base learner, effectively pooling their collective knowledge to generate a consensus prediction. By averaging the predictions, AvgVotReg aims to reduce the impact of individual errors or biases inherent in any single model. The ensemble approach of AvgVotReg offers several advantages. Firstly, it helps to mitigate overfitting by leveraging the diversity of models. Since each base learner may have different strengths and weaknesses, combining them can lead to more stable and generalizable predictions. Additionally, ensemble methods often perform better than individual models by reducing variance and bias in the predictions.

AvgVotReg harnesses the complementary strengths of RF, XGBoost, and ANN through an ensemble approach, resulting in improved prediction performance for the regression task. By combining the predictions of multiple models, AvgVotReg aims to achieve robust and accurate predictions that outperform any individual model alone.

### 6.5. Two-Level Sequential Cascade Generalization (2LSCG)

The 2LSCG architecture is a structured model that operates in two distinct levels: classification and regression. This sequential model is designed to address tasks that involve both classification and regression components, such as certain predictive modeling problems. In the first level of the architecture, the input data are processed through a classifier. This classifier is responsible for assigning class labels or probabilities to the input samples based on their features. The classification probabilities generated by the classifier serve as intermediate representations of the input data, capturing information relevant to the classification task. In the second level, referred to as Regressor-1, the classification probabilities obtained from the first level are utilized as features for a regression task. These probabilities serve as informative signals that are fed into the regression model to predict continuous target variables or quantities of interest.

**Figure 4:**
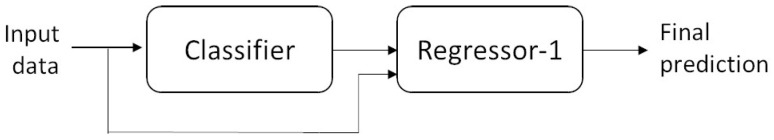
Two-level sequential cascade generalization (2LSCG) architecture.

The sequential nature of the architecture implies that the output of the classifier acts as input to the regression model. This hierarchical arrangement allows the regression model to leverage the informative features extracted by the classifier, potentially improving the accuracy and robustness of the regression predictions. The two-level structure of the 2LSCG architecture offers several advantages. By separating the classification and regression tasks into distinct levels, the model can effectively handle complex predictive tasks that involve both classification and regression components. Additionally, the use of intermediate classification probabilities as features for regression enables the model to capture nuanced relationships between the input data and the target variables. Figure 4 depicts the architecture of 2LSCG.

### 6.6. Three-Level Sequential Cascade Generalization (3LSCG)

The 3LSCG architecture is an extended version of the 2LSCG architecture, featuring an additional regression level. This sequential model consists of three distinct levels: classification, Regressor-1, and Regressor-2. Each level performs specific tasks in a sequential manner, allowing the model to address complex predictive tasks that involve multiple stages of processing. In the first level, the input data are processed through a classifier, similar to the initial level of the 2LSCG architecture. The classifier assigns class labels or probabilities to the input samples based on their features, generating outputs that represent the classification probabilities for each class. The output probabilities of the classifier serve as inputs to the second level, referred to as Regressor-1. In addition to the classifier probabilities, the input data themselves are also included as input to Regressor-1. This combination of inputs allows Regressor-1 to leverage both the informative features extracted by the classifier and the raw input data to perform the first stage of regression. The output of Regressor-1, which represents the predicted regression values based on the inputs from the classifier and the raw data, serves as input to the third level, known as Regressor-2. Similar to Regressor-1, Regressor-2 receives both the output of Regressor-1 and the original input data. This hierarchical arrangement enables Regressor-2 to refine the regression predictions further, incorporating information from both the intermediate regression outputs and the raw input features.

**Figure 5:**
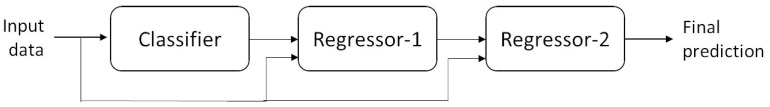
Three-level sequential cascade generalization (3LSCG) architecture.

By organizing the predictive tasks into multiple levels of processing, the 3LSCG architecture offers a hierarchical and modular approach to predictive modeling. Each level focuses on specific aspects of the predictive task, allowing the model to capture complex relationships and dependencies within the data. Additionally, the sequential nature of the architecture enables the model to iteratively refine its predictions, potentially improving the accuracy and robustness of the final outputs. Figure 5 depicts the architecture of 3LSCG.

### 6.7. Parallel Cascade Generalization (PCG)

The PCG architecture presents a parallelized approach to predictive modeling, featuring two concurrent levels of processing followed by a final regression level. This parallel model is designed to leverage computational resources efficiently and expedite the predictive task by executing multiple stages simultaneously. At the first level, the architecture simultaneously performs classification and regression tasks. This level comprises two components: first-level classification and regression, denoted as Regressor-1. These components operate concurrently, allowing the model to process input data in parallel streams. The first-level classification component classifies input samples into predefined classes or assigns probabilities to each class, while the Regressor-1 component simultaneously generates regression predictions based on the input data. The outputs of the first-level classification and Regressor-1 are then fed into the final level, referred to as Regressor-2. In addition to these intermediate outputs, the input data are also included as input to Regressor-2. This level integrates the classification probabilities and regression predictions from the previous stages with the raw input features, enabling the model to refine its predictions further and capture complex relationships within the data.

**Figure 6:**
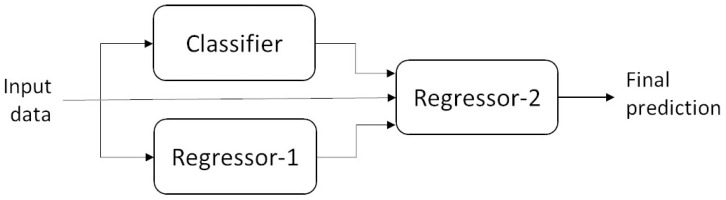
Parallel cascade generalization (PCG) architecture.

Figure 6 illustrates the architecture of PCG, depicting the parallel streams of processing at the first level and the integration of outputs at the final level. This visualization highlights the concurrent execution of classification and regression tasks at the first level and the sequential refinement of predictions at subsequent stages. By leveraging parallel processing capabilities, the PCG architecture offers advantages in terms of computational efficiency and speed, particularly for tasks involving large datasets or complex predictive models. The parallel execution of classification and regression tasks allows the model to exploit available resources effectively, leading to improved scalability and performance.

## 7. Experimental Protocols and Evaluation Measures

The models in this study were implemented using the TensorFlow 2.6.0 [45] framework, along with the Keras [46], Pandas [47], Scikit-Learn [48], and SciPy [49] libraries. The implementation was carried out on a Jupyter Notebook Server 6.4.6 with Python 3.8.5. The hardware for the implementation included a laptop with M1 CPU and 8 GB system memory.

### 7.1. Model Selection

For this study, we employed a five-fold nested cross-validation (CV) procedure [50]. Hyper-parameters were selected using the RandomizedSearchCV method [48]. A nested CV estimates the generalization error of the base model in its hyper-parameter search to prevent over-fitting and ensure model accuracy and reliability.

In the subsequent paragraphs, we outline the parameters and parameter ranges for all of the algorithms utilized in this study. The default parameter values were sourced from the Scikit-Learn website.

The RF classifier and regressor in this study were subjected to hyper-parameter tuning. The candidate parameters were as follows: The number of trees ranged from 50 to 1000 in increments of 50, the maximum number of features to consider at each split was set to auto or sqrt, and the maximum number of levels in the tree was limited to 31. Default values were employed for all other hyper-parameters.

The ANN classifier in this study underwent hyper-parameter tuning with the following candidate parameters: The number of nodes in each hidden layer ranged from 5 to 150 in increments of 5, and the learning rates were selected from the values 0.1, 0.01, 0.001, or 0.0001. A dropout layer was added to each hidden layer with three different rates (i.e., 10%, 30%, or 50%). Four activation functions were utilized—tanh, swish, linear, and ReLU—with the optimizer set as ADAM and the loss function defined as mean absolute error.

The XGBoost regressor in this study underwent hyper-parameter tuning with the following candidate parameters: The number of trees in the ensemble ranged from 50 to 1000 in increments of 50, the maximum number of levels in the tree ranged from 1 to 31, and the learning rates were 0, 0.51, and 0.01. The sub-sample rates for rows and columns used in each tree varied from 0.0 to 1.0 in increments of 0.05. The loss functions utilized were squared error, logistic, or absolute error. Default values were employed for all other hyper-parameters.

### 7.2. Performance Measures

To assess the performance of the models, we employ diverse evaluation metrics, such as accuracy (Acc) for classification tasks and R-squared ($R^{2}$), mean absolute error (MAE), mean squared error (MSE), and root mean squared error (RMSE) for regression tasks.

Let TP and TN denote the numbers of true positives and true negatives, respectively, and FP and FN denote the numbers of false positives and false negatives, respectively. Acc is defined as the proportion of correctly predicted examples (1).

(1) $$Acc=\frac{TP+TN}{TP+TN+FP+FN}.$$

Let $y_{i}$ represent the predicted value for the ith observation and $\hat{y_{i}}$ represent the actual value. Considering *N* observations with mean observed value $\bar{y}$, MAE evaluates the average absolute deviation between actual and predicted data (2):

(2) $$MAE=\frac{\sum_{i=1}^{N}\left|y_{i}-\hat{y_{i}}\right|}{N}.$$

The MSE measures the average squared variance between observed data and predicted data (3):

(3) $$MSE=\frac{\sum_{i=1}^{N}{\left(y_{i}-\hat{y_{i}}\right)}^{2}}{N}.$$

$R^{2}$ is a statistical measure that quantifies the proportion of total variance in the dependent variable that is attributable to the independent variables. Its value ranges between 0 (indicating that the model explains none of the variance) and 1 (indicating that the model accounts for all of the variance) (4):

(4) $$R^{2}=1-\frac{\sum_{i=1}^{N}{\left(y_{i}-\hat{y_{i}}\right)}^{2}}{\sum_{i=1}^{N}{\left(y_{i}-\bar{y}\right)}^{2}}.$$

The RMSE metric quantifies the degree of agreement between predicted values and actual results (5):

(5) $$RMSE=\sqrt{\frac{\sum_{i=1}^{N}{\left(y_{i}-\hat{y_{i}}\right)}^{2}}{N}}.$$

## 8. Results

In Section 6 of this study, we defined seven distinct architectures, comprising three regressors and four ensemble architectures. To select the hyper-parameters outlined in Section 7.1, we utilized RandomSearchCV. The regressors employed in this research were RF, XGBoost, and a two-layer ANN, with their respective outcomes presented in Section 8.1. Additionally, Section 8.2 reports the results of the random forest classifier, while the ensemble architectures are detailed as follows: AvgVotReg in Section 8.3, 2LSCG in Section 8.4, 3LSCG in Section 8.5, and PCG in Section 8.6.

### 8.1. Results of Learning Machines

**Figure 7:**
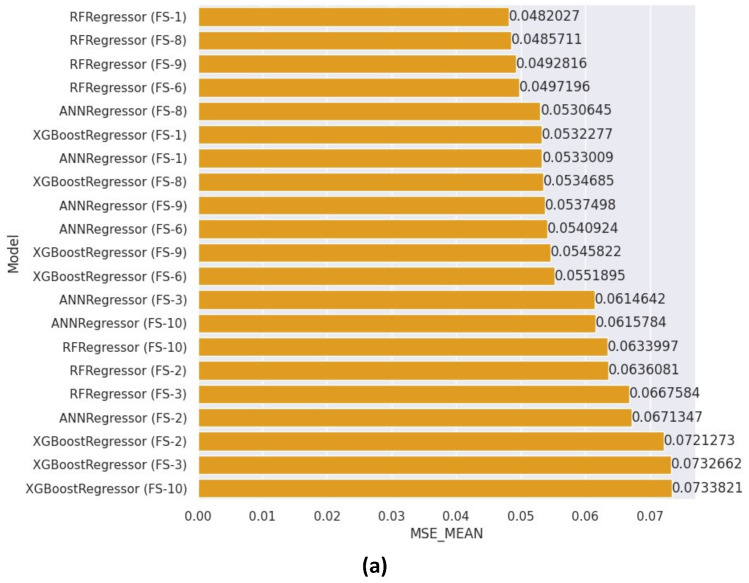

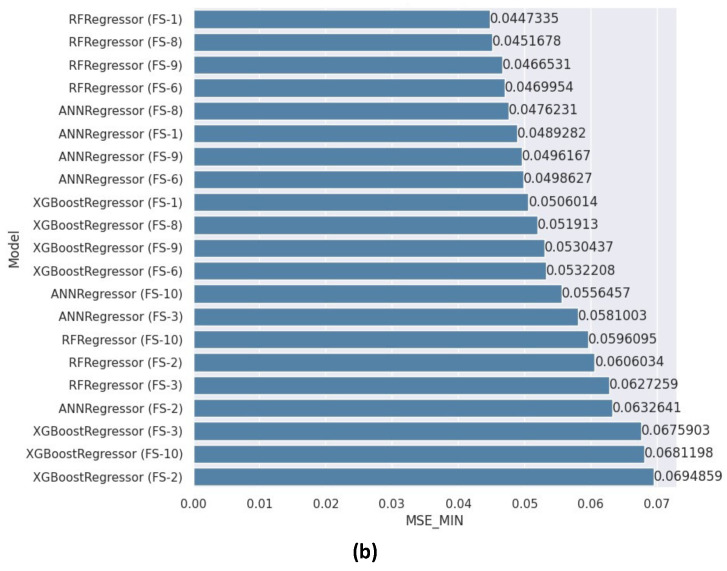
Performance scores for the independent learning machines: (**a**) The mean MSE achieved by the different learning machines; and (**b**) minimum MSE achieved by the different learning machines.

The mean MSE values obtained by different independent learners are presented in Figure 7a, with the feature subsets used for each model labeled on the left side of the figure (as defined in Table 3). For instance, RandomForestRegressor (FS-1) refers to a random forest regression learner utilizing all features, while XGBRegressor (FS-10) denotes an XGBoost regression learner employed in conjunction with features selected through the chi-square and Kruskal–Wallis tests. As indicated by Figure 7a, the RandomForestRegressor employing all features outperformed the other models, achieving a mean MSE of 0.0482. In contrast, the XGBoost regressor utilizing the feature subset obtained from the chi-square and Kruskal–Wallis tests attained the highest mean MSE of 0.0733. Figure 7b displays the minimum MSE scores achieved by the different independent models. As illustrated by the figure, the random forest regressor (RandomForestRegressor) employing all features outperformed the other models, attaining a minimum MSE of 0.0447. In contrast, the least successful model scored 0.0695. The hyper-parameters selected by the RandomSearchCV algorithm for the RandomForestRegressor (FS-1) model consisted of 800 N-estimators, Auto as the maximum features setting, and a maximum depth of 28.

### 8.2. Classifying Patients According to Their LOS Patterns

**Figure 8:**
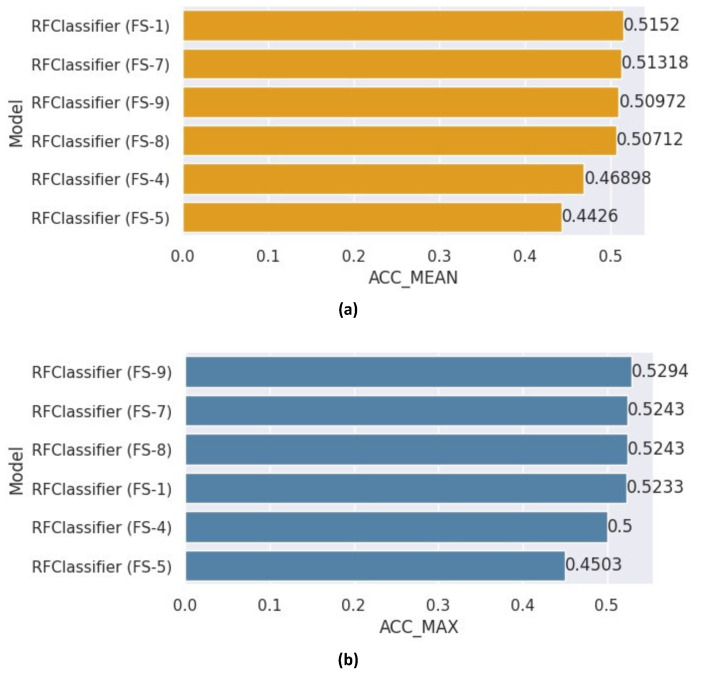
Performance scores for the different RF classifier models using different feature subsets: (**a**) the mean Acc scores for the different RF classification models; (**b**) the maximum Acc scores for the different RF classification models.

Figure 8 depicts the mean and maximum accuracy scores attained in various experiments utilizing different feature sets for the RF classifier. Regarding mean accuracy, the experiment utilizing FS-1 outperformed the others, achieving an accuracy of 0.5152. On the other hand, for maximum accuracy, the experiment utilizing FS-9 scored the highest accuracy of 0.5294. The optimal hyper-parameters for the RF classifier, as selected by RandomSearchCV, were as follows: Random-state of 42, N-estimators set to 550, max-features designated as Auto, and max-depth of 29. These parameters were used for all subsequent ensemble models utilizing the RF classifier.

**Figure 9:**
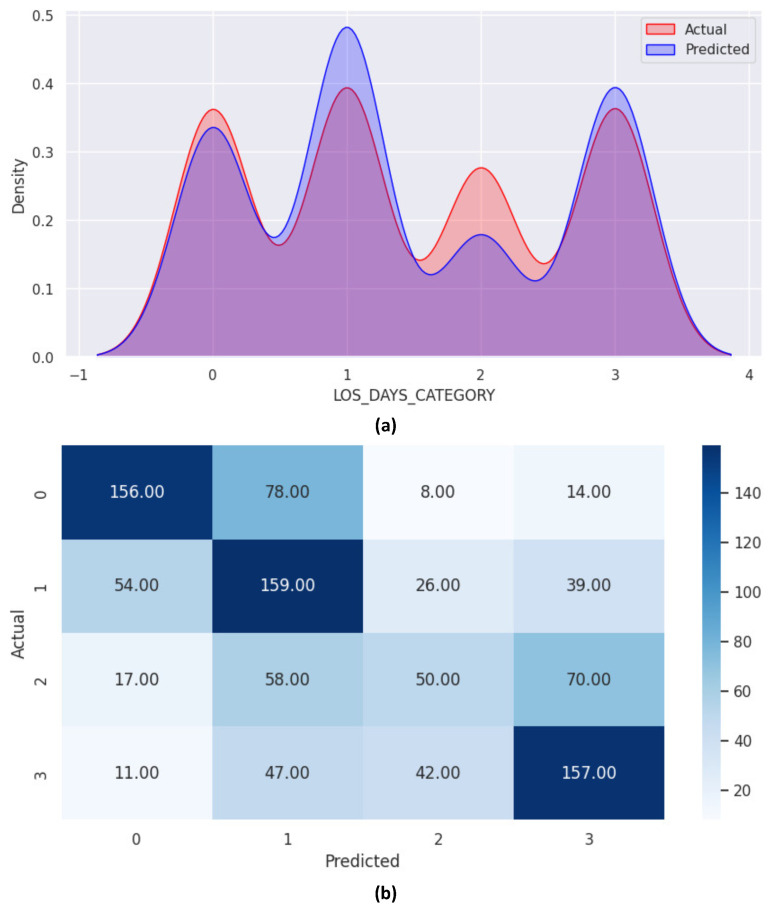
Actual versus predicted LOS-DAYS-CATEGORY showing the performance of the RF classifier in the LOS-DAYS classification task: (**a**) density graph for the LOS-DAYS-CATEGORY; (**b**) confusion matrix.

Figure 9a presents the density graph for LOS-DAYS-CATEGORY, revealing the most significant discrepancy between actual and predicted categories for Class 1 ([3–6] days) and Class 2 ([7–11] days). The confusion matrix, illustrated in Figure 9b, provides further insights. As indicated in Figure 9b, the diagonal elements of the confusion matrix correctly classified 522 instances.

### 8.3. Average Voting Regression (AvgVotReg) Ensemble Results

**Figure 10:**
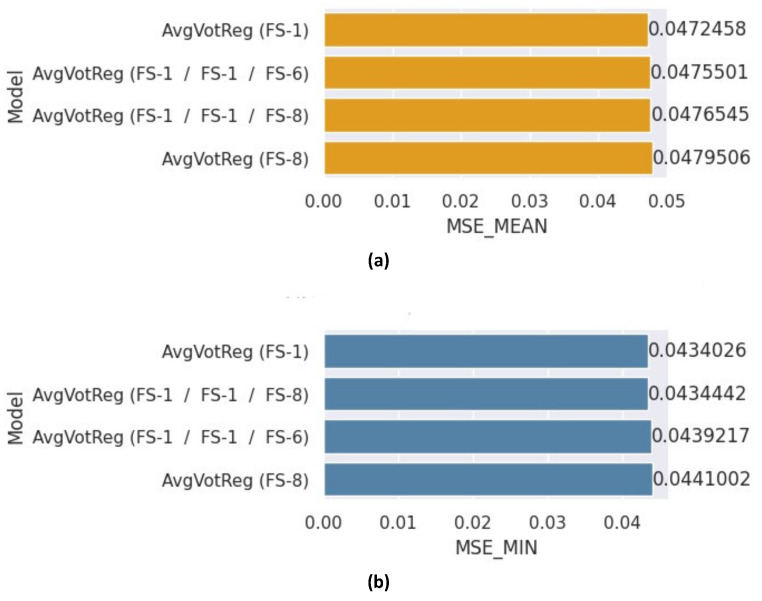
Performance scores for the AvgVotReg ensemble of three regressors (RF, XGBoost, and ANN) utilizing different feature subsets: (**a**) Mean MSE scores for the different AvgVotReg ensemble models; (**b**) minimum MSE scores for the different AvgVotReg ensemble models.

An ensemble model was developed by averaging the predictions of RF, XGBoost, and ANN regressors. Each individual regressor was trained using distinct feature subsets, as shown in Figure 10. The first ensemble involved all models utilizing all features, while the second ensemble consisted of RF and XGBoost with all features selected and the ANN with FS-6. The third ensemble included RF and XGBoost with all features and ANN with FS-8, and the final ensemble employed all models with FS-8. The mean and minimum MSE scores achieved by these ensembles are presented in Figure 10a,b, respectively. Notably, the ensemble model reporting the average predictions from regressors utilizing all features outperformed the other models, attaining a mean MSE of 0.0472 and a minimum MSE of 0.0434. Conversely, the weakest ensemble was the last, utilizing features selected according to the Kruskal–Wallis test (FS-8).

**Figure 11:**
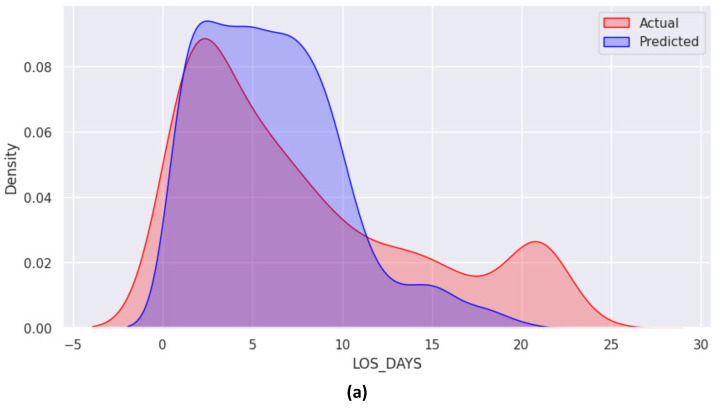

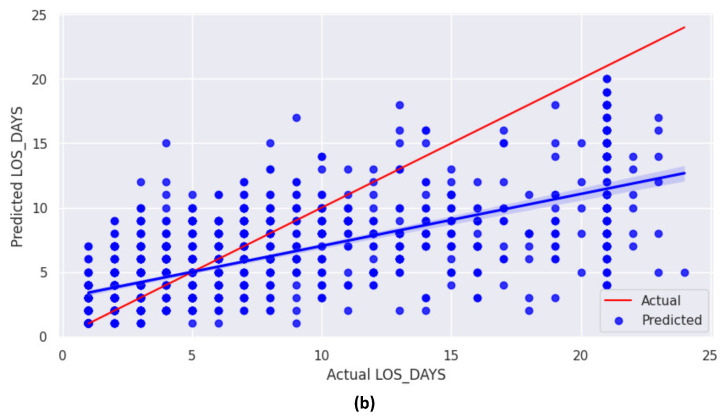
Actual versus predicted LOS-DAYS-CATEGORY showing the performance of the AvgVotReg ensemble in the LOS-DAYS prediction task: (**a**) density graph for LOS-DAYS; (**b**) linear graph for actual versus predicted LOS-DAYS.

The RandomedSearchCV method selected the optimal hyper-parameters for the XGBoost regressor of the AvgVotReg (FS-1) model as follows: sub-sample rate = 0.95, N-estimators = 500, reg:logistic as an objective, maximum depth = 17, and colsample-bytree = 0.8. On the other hand, the optimal hyper-parameters for the ANN regressor were a learning rate of 0.01, dropout rates of 0.1 in both layers, and Swish and ReLU as activation functions in hidden layers 1 and 2, respectively. The input shape for the ANN was 77, and 5 and 70 neurons were included in the first and second hidden layers, respectively. Figure 11a,b exhibits the density graph for LOS days and the actual versus predicted LOS days, respectively. As demonstrated in Figure 11, LOS-DAYS had a positively skewed distribution with a peak value at 2 days and a median value at 7 days, representing the center of the distribution. The models provided reasonably accurate predictions for LOS-DAYS ranging from 0 to 3 days and 11 to 13 days; however, there was a significant disparity for LOS-DAYS within the range of 5–10 days.

### 8.4. Two-Level Sequential Cascade Generalization (2LSCG) Results

**Figure 12:**
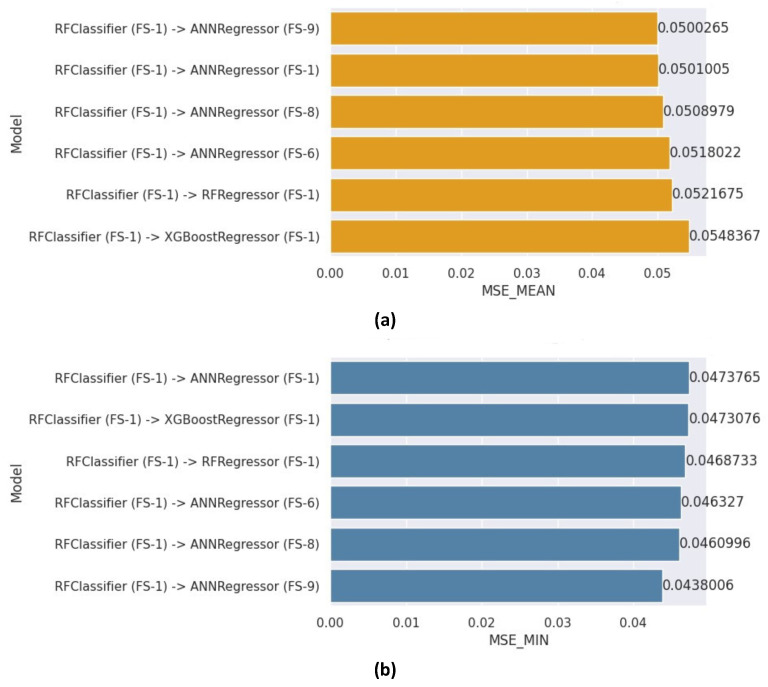
Performance scores of the 2LSCG models utilizing different feature subsets: (**a**) mean MSE scores for the different 2LSCG models; (**b**) minimum MSE scores for the different 2LSCG models.

The two-level stacked cascade generalization (2LSCG) comprised an RF classifier followed by a regressor. Figure 12a,b presents the mean and minimum MSE scores attained by the various 2LSCG models, respectively. The *y*-axis denotes the models, where the identifier after the “−>” sign specifies the learner and feature subset adopted for the regressor component of the cascade generalizer; for instance, in the first model, “RFclassifier (FS-1) −> ANN (FS-9),” the RF classifier utilizing FS-1 is succeeded by an ANN employing FS-9. Among all the models, the model utilizing the FS-1 feature subset for the RF classifier and FS-9 for the ANN regression learner outperformed the other models, achieving a mean MSE of 0.0500 and a minimum MSE score of 0.0438.

As determined by the RandomSearchCV method, the optimal hyper-parameters for the ANN base learner were a learning rate of 0.01 and a dropout rate of 0.1 in both of the ANN layers. The activation functions were Swish and ReLU in the first and second layers, respectively. The input shape for the ANN was 81, and 5 and 70 neurons were included in the first and second hidden layers, respectively.

### 8.5. Three-Level Sequential Cascade Generalization (3LSCG) Results

**Figure 13:**
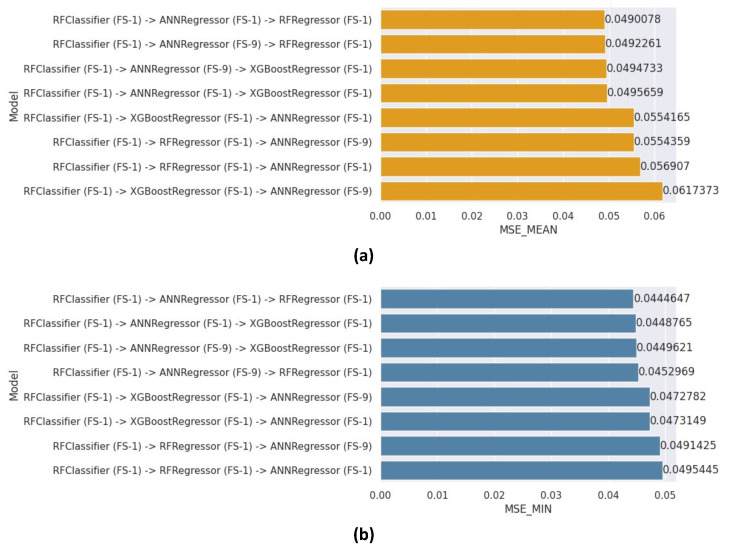
Performance scores of the 3LSCG models utilizing different feature subsets: (**a**) mean MSE scores for the different 3LSCG models; (**b**) minimum MSE scores for the different 3LSCG models.

Six ensemble models were created for the 3LSCG architecture, utilizing various combinations of learners in the various levels (Classification, Regression-1, and Regression-2). Figure 13a,b displays the mean and minimum MSE values achieved by these models, respectively. The model utilizing the RF classifier with FS-1, Regression-1 utilizing the ANN regressor, and Regression-2 employing the RF regressor with FS-1 outperformed the other models, attaining a mean MSE of 0.0490 and a minimum of 0.0445. Conversely, the weakest model was the one utilizing an RF classifier with FS-1, Regression-1 using XGBoost regressor with FS-1, and Regression-2 employing the ANN regressor with FS-9, with a mean MSE of 0.0617. Additionally, the ensemble using the RF classifier with FS-1, Regression-1 utilizing the RF regressor with FS-1, and Regression-2 employing the ANN regressor with FS-1 was the worst-performing model in terms of minimum MSE, with a score of 0.0495.

In the best-performing 3LSCG model, the optimal hyper-parameters utilized for the RF component were as follows: N-estimators of 550, max-features set to Auto, and a max depth of 29. Interestingly, the architecture and best hyper-parameters for the ANN component were found to be identical to those employed in the best-performing 2LSCG model.

### 8.6. Parallel Cascade Generalization (PCG) Results

**Figure 14:**
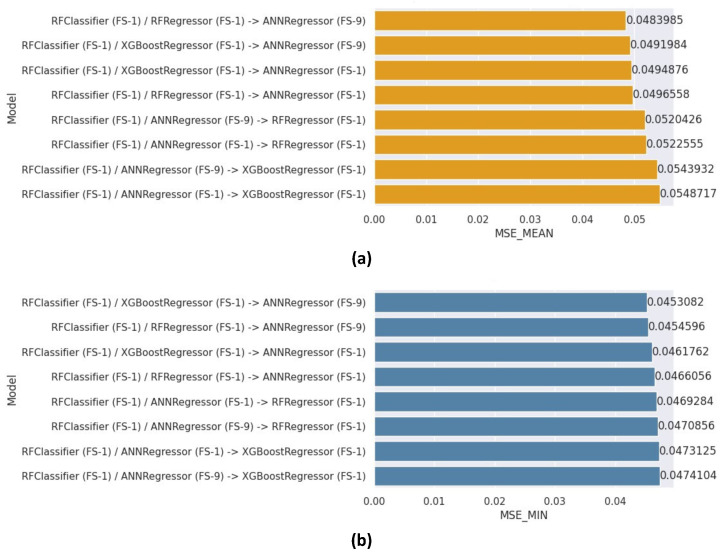
Performance scores of the PCG models utilizing different feature subsets: (**a**) mean MSE scores for the different PCG models; (**b**) minimum MSE scores for the different PCG models.

Eight ensemble models were created for the PCG architecture, utilizing various combinations of learners at the various levels (Classification, Regression-1, and Regression-2) and feature subsets in each component. Figure 14a,b depicts the mean and minimum MSE values obtained by these models, respectively. The model utilizing the RF classifier with FS-1, Regression-1 employing the RF regressor with FS-1, and Regression-2 utilizing the ANN regressor with FS-9 outperformed the other models, attaining a mean MSE of 0.0484 and minimum MSE of 0.0453. Conversely, the lowest performance was achieved by an RF classifier with all features, an ANN regressor (all features), and the last level as an XGBoostRegressor (all features), achieving a mean MSE of 0.0549.

The best hyper-parameters for the ANN component were again found to be identical to those employed in the best-performing 2LSCG model.

## 9. Discussion

For this study, we considered three basic ML approaches for the prediction of LOS—namely, the RF regressor, XGBoost regressor, and ANN regressor—as well as four ensemble architectures: 2LSCG, 3LSCG, AvgVotReg, and PCG. Table 4 presents a comparison of the best models among all the architectures, where the best MSE scores are highlighted in bold typeface. Among the independent models, the RF regressor with FS-1 achieved the lowest mean MSE (0.0482) and minimum MSE (0.0447), while XGBoost had the highest mean MSE (0.0733) and minimum MSE (0.0694). Similar conclusions can be drawn considering the RMSE. In contrast, the best-performing ensemble architecture as measured by the MSE was the average voting regression utilizing all features, which outperformed other models, with a mean MSE of 0.0472 and minimum MSE of 0.0434. Regarding the assessment of prediction errors, we have considered mean absolute error (MAE) as our primary metric. Our analysis reveals interesting distinctions among the models. XGBoost, among the independent regressors, exhibits superior performance, indicating that it provides more accurate predictions on average when compared to other independent models. Within the cascaded generalizer category, 3LSCG outperforms its counterparts in terms of MAE. Furthermore, when comparing AvgVotReg and 3LSCG, it is evident that AvgVotReg excels in fitting outliers, patients with unusually long or short stays, but lags behind when dealing with patients whose stays fall within the typical range.

While evaluating the performance of various models in predicting patient LOS, we explored a unique approach involving patient classification based on data patterns. This approach allowed us to employ different learning models tailored to each patient group efficiently. One of the classifiers we used at the first level was the RF classifier. Interestingly, we observed that the use of the RF classifier at this initial stage had a noticeable impact on subsequent levels, particularly in the 2LSCG and 3LSCG cascading architectures, as well as in the PCG architecture.

The observed impact could be attributed to the sequential nature of the cascading design. In this design, the results of the RF classifier serve as inputs to another learner at the second level. While this design introduces the potential for enhanced modeling, it also carries the risk of introducing noise or misclassifying certain classes, which can affect subsequent predictions.

To visualize these challenges, we examined differences between observed and actual LOS days using a density plot (Figure 9a). This analysis revealed specific challenges faced by the RF classifier, including misclassification of Class 1 within the three to six days period and Class 2 within the seven to eleven days period.

Among all the models evaluated, the AvgVotReg utilizing the FS-1 feature set achieved the lowest mean MSE (0.0472) and minimum MSE (0.0434), outperforming the other architectures. This is because the AvgVotReg combines the outputs of three separate basic regressors, allowing the strong learner(s) to compensate for the poor prediction of the weak learner(s). Additionally, the RF regressor, utilizing random sampling of training data and features, generates multiple decision trees whose predictions are averaged to obtain a final prediction, thereby mitigating over-fitting and enhancing model performance. Ensembling is an in-house function in RF [51].

**Figure 15:**
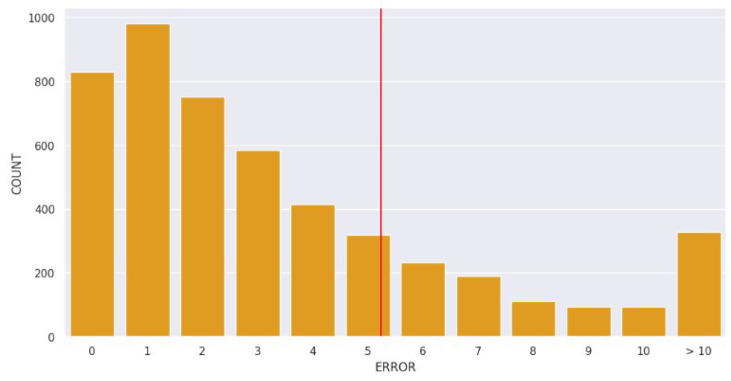
Error observed for AvgVotReg model.

A more thorough evaluation of the error for the model named AvgVotReg was conducted. A graph depicting the error is presented in Figure 15, which was calculated by subtracting the predicted LOS days from the actual LOS days. The graph indicates that 800 instances were correctly predicted, and the majority of errors for predicted values were within one day of the actual LOS. As the difference in the number of days between the predicted and actual LOS increased, fewer errors were observed.

**Figure 16:**
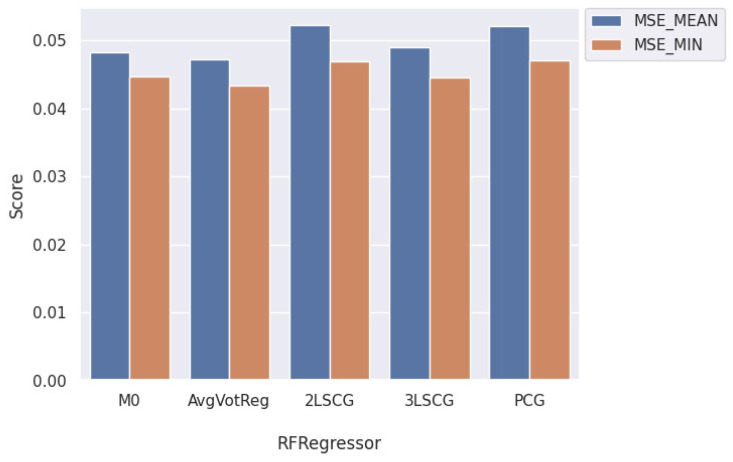
Performance comparison of the best-performing architectures utilizing a random forest regressor.

Figure 16 presents a performance comparison of the best-performing models utilizing RF regression learners in their architecture, with M0 representing the basic RF regressor. Among all the proposed architectures, AvgVotReg outperformed the others. However, there was a discernible decline in the performance of the RF in both the 2LSCG and PCG architectures, which could be attributed to the negative effect of the RF classifier results on these two architectures.

**Figure 17:**
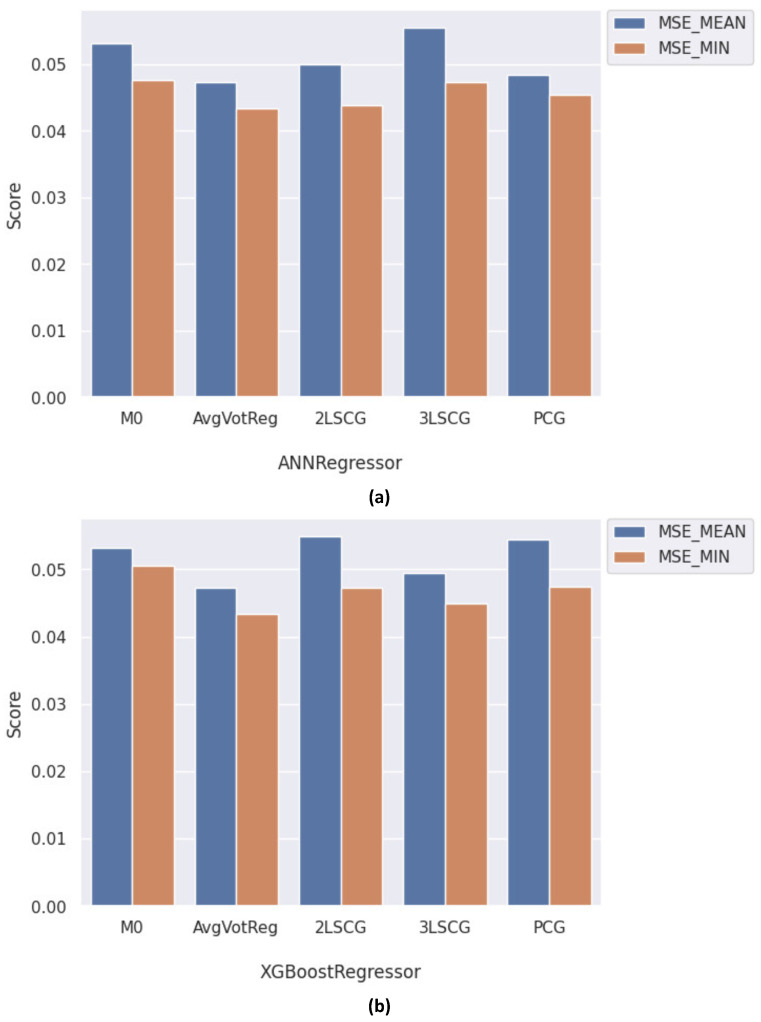
Performance comparison of the best-performing architectures utilizing (**a**) an ANN regressor and (**b**) an XGBoost regressor.

Figure 17a shows a comparison of the best-performing models utilizing ANN regression learners in their architecture, with M0 representing the basic ANN regressor. Notably, the performance of the ANN learner declined in the 3LSCG architecture, due to the propagation of errors through the layers, resulting in double the error of both the 2LSCG and PCG architectures. Among the proposed architectures, AvgVotReg emerged as the top-performing model, outperforming the others. A similar observation can be drawn from Figure 17b, which provides a performance comparison of the best-performing models utilizing the XGBoost regression learner among all architectures, with M0 representing the basic XGBoost regressor.

The success of cascading ensembles in parallel is dependent on several factors, including the competence of the individual learners and the usefulness of the information passed between them.

From an algorithmic standpoint, the performance comparison among various machine learning models, as depicted in Table 4, offers insights into their suitability for the patient LOS regression task. RF, known for its ensemble learning approach, exhibits robust performance across multiple metrics, showcasing its ability to capture complex relationships in the data effectively. XGBoost, renowned for its scalability and optimization techniques, demonstrates competitive performance albeit slightly lower than RF in terms of predictive accuracy. ANNs, although versatile in capturing intricate patterns, show comparable performance with higher variability, indicating the necessity for meticulous hyperparameter tuning and regularization to prevent overfitting and achieve optimal results. On the other hand, AvgVotReg emerges as a top-performing model, leveraging ensemble learning principles to combine predictions from multiple base models effectively. Its superior performance across $R^{2}$, MSE, RMSE, and MAE underscores the efficacy of ensemble techniques in enhancing predictive accuracy and reducing model variance. Additionally, cascade generalization models (2LSCG, 3LSCG, PCG) introduce a novel approach by integrating dedicated LOS classifiers within sophisticated ensemble methodologies. While these models demonstrate competitive performance, particularly 3LSCG in terms of MAE, their algorithmic complexity and interpretability may pose challenges, necessitating careful consideration of trade-offs between performance and complexity in real-world deployment.

## 10. Limitations, Implications, and Recommendations for Practical Deployment of ML-Based LOS Prediction

While extensive research has been conducted on LOS prediction, with promising experimental results reported in the literature, practical deployment constraints have received relatively little attention. Many studies lack real-world validation of trained machine-learning models and fail to discuss the various ways in which such models can be utilized. In this section, we emphasize significant limitations, implications, and recommendations regarding the deployment of ML-based solutions.

A few of the highlighted limitations in developing end-to-end practical solutions for LOS prediction in clinical settings are listed as follows:

- Data quality assurance: While ML models depend on high-quality data for accurate predictions, healthcare data often suffer from inconsistencies, missing values, and errors. Incomplete or inaccurate data entries can significantly impact the performance and reliability of LOS prediction models. Implementing robust data quality assurance measures, such as data cleaning, normalization, and validation, is crucial to mitigate this limitation and ensure the integrity of the predictive models.
- Clinical interpretability: Many ML algorithms, particularly complex deep learning models, lack interpretability, making it challenging for healthcare practitioners to understand the rationale behind model predictions. Clinicians require transparent and interpretable models to trust and effectively utilize the predictions in clinical decision-making. Developing explainable AI techniques tailored to healthcare contexts can help address this limitation by providing clinicians with insights into the factors influencing LOS predictions.
- Integration with clinical workflows: Successful deployment of ML-based LOS prediction models hinges on their seamless integration into existing clinical workflows. However, integrating ML models into complex healthcare systems poses technical and logistical challenges. Healthcare institutions must invest in interoperable and user-friendly systems that facilitate the seamless integration of ML models into clinical decision-support tools. Collaborating closely with healthcare IT professionals and stakeholders is essential to ensure the smooth adoption and usability of ML-based solutions in clinical settings.
- Ethical and regulatory compliance: Healthcare data used for training ML models are subject to stringent privacy regulations and ethical considerations. Ensuring compliance with data privacy laws, while facilitating data sharing and model deployment for research and clinical purposes, is paramount. Implementing robust data anonymization, encryption, and access control mechanisms is necessary to protect patient privacy and maintain regulatory compliance throughout the model development and deployment process.

Trained ML models for predicting LOS offer various avenues for improving healthcare facility operations. Below are some of the key implications of employing LOS prediction ML models:

- Clinical decision support: ML-based LOS prediction models can serve as valuable decision support tools for clinicians, offering insights into patient LOS and risk stratification. By providing clinicians with actionable predictions, these models empower healthcare providers to optimize patient flow, allocate resources effectively, and make informed discharge decisions, ultimately improving patient outcomes and enhancing the quality of care.
- Operational efficiency: Leveraging ML-based LOS prediction models can enhance operational efficiency in clinical settings by streamlining patient management processes and optimizing resource allocation. Hospitals can use predictive insights to improve throughput, reduce wait times, and enhance patient satisfaction by minimizing unnecessary delays in discharge planning and optimizing workflow management.
- Resource allocation and staffing optimization: ML-based LOS prediction models enable hospitals to accurately forecast patient volumes and acuity levels, facilitating proactive resource allocation and staffing optimization. By anticipating patient needs and adjusting staffing levels accordingly, hospitals can ensure timely and efficient patient care delivery, mitigate overcrowding, and optimize resource utilization to meet patient demand effectively.

Despite significant technological advancements and available training resources, integrating ML technologies into established clinical frameworks has encountered challenges, and trust in ML-based predictions remains uncertain. Some recommendations to enhance the practicality and deployability of ML-based solutions include:

- Continuous model evaluation and improvement: ML-based LOS prediction models should undergo continuous evaluation and improvement to maintain relevance and performance in dynamic clinical environments. Regular model monitoring, feedback collection from clinicians, and iterative model refinement based on real-world performance data are essential for ensuring the accuracy and reliability of predictions over time.
- Clinician training and education: Providing comprehensive training and education to healthcare professionals on the interpretation and utilization of ML-based LOS prediction models is essential for successful deployment. Clinicians should receive training on understanding model outputs, interpreting prediction uncertainties, and effectively integrating model insights into clinical decision-making processes. Ongoing education programs and workshops can help keep clinicians abreast of the latest developments in ML-based healthcare technologies and their practical applications.
- Interdisciplinary collaboration: Foster interdisciplinary collaboration between data scientists, clinicians, healthcare administrators, and IT professionals to facilitate the development, deployment, and adoption of ML-based LOS prediction solutions. Collaborative efforts ensure that ML models are aligned with clinical needs, seamlessly integrated into existing workflows, and effectively utilized to improve patient care outcomes. Establishing multidisciplinary teams dedicated to developing and implementing ML-based solutions can foster innovation, knowledge exchange, and successful technology adoption in healthcare settings.
- Transparency and communication: Maintain transparent communication channels with patients, clinicians, and stakeholders regarding the use of ML-based LOS prediction models in clinical settings. Providing clear explanations of model functionalities, limitations, and potential biases fosters trust, promotes acceptance, and enhances the ethical and responsible deployment of ML solutions in healthcare settings. Open dialogue and engagement with stakeholders can help address concerns, dispel misconceptions, and foster a culture of transparency and accountability in the deployment of ML-based healthcare technologies.

## 11. Conclusions

In this study, we focused on predicting the LOS for cardiac patients during their hospitalization, utilizing a proprietary dataset and a range of ML techniques. Accurate LOS predictions hold the potential to support healthcare professionals in making informed decisions and optimizing the allocation of hospital resources. To achieve this goal, we developed and assessed seven distinct models, encompassing both individual models such as RF, XGBoost, and ANN, as well as ensemble models including 2LSCG, 3LSCG, PCG, and AvgVotReg. The research considered the LOS prediction as a continuous variable problem instead of a categorical one. A novel proprietary dataset referred to as SaudiCardioStay (SCS) was developed based on the data of adult cardiac patients from KFSH&RC hospital, a leading healthcare institution in Saudi Arabia. Among the tested algorithms, RF demonstrated strong performance, achieving a mean MSE of 0.0482. XGBoost and ANNs followed closely, with mean MSE values of 0.0532 and 0.0530, respectively. Notably, the ensemble technique AvgVotReg emerged as the most effective, achieving the lowest mean MSE of 0.0472. Additionally, cascade generalization models exhibited promising results, with 3LSCG achieving a mean MSE of 0.0490. The PCG architecture also demonstrated competitive performance, with a mean MSE of 0.0483. Based on the MAE from the experimental results, the 3LSCG model emerged as the top-performing with the lowest value of 0.1700. However, the AvgVotReg performed comparatively, with an MAE of 0.1706. These findings underscore the potential of ensemble methods and cascade generalization models in improving predictive accuracy for patient LOS regression tasks. The clinical validation of the trained ML models is one of the potential future works of this research. Furthermore, the research could benefit from the latest generative AI technologies for creating efficient simulated datasets which can significantly help train the ML models toward better performance. 

## Figures and Tables

**Table 1:** Full initial feature set showing the domain for each categorical feature, the percentage of missing values (PMV), the type as categorical (Cat.), numerical (Num.), or date/time, and the source dataset.

I	Feature	Description	Domain	PMV	Type
1	SEX	Sex of patient.	2	0	Cat.
2	ADMIT-TYPE	The type of admission, such as ‘Emergency’, ‘Scheduled’, ‘Direct admission’.	5	0	Cat.
3	ADMIT-MODE	Mode of hospital admission, such as ‘Wheelchair’, ‘KFSH Ambulance’.	11	0	Cat.
4	NURSE-UNIT	The name of the nurse unit.	74	0	Cat.
5	SOURCE-STRING	Diagnosis on admission, such as ‘Bradycardia’, ‘Mitral stenosis’.	3193	0	Cat.
6	ADMISSION-ARRIVE-DT-TM	The arrival date for the current admission.	[1 January 2019, 1 January 2023]	0	Datetime
7	TASK-ASSAY	The name of the lab test, such as ‘Creat’, ‘Na’, ‘RBC’, ‘Urea’, ‘WBC’.	27	0	Cat.
8	RESULT-VALUE-NUMERIC	The results of lab tests of TASK-ASSAY.	[0, 70,000]	0.004	Num.
9	PRIOR-ADMISSION-ARRIVE	Arrival date for prior admission.	[1 January 2018, 31 December 2022]	0.285	Datetime
10	PRIOR-ADMISSION-DISCHARGE	The prior admission discharge date.	[1 January 2018, 31 December 2022]	0.285	Datetime
11	DISCH-DT-TM	The date of the patient’s discharge following their previous surgical procedure.	[1 January 2018, 14 January 2023]	0	Datetime
12	ARRIVE-DT-TM	The date of the patient’s arrival for their previous surgical procedure.	[1 January 2018, 12 January 2023]	0	Datetime
13	Oxygen Saturation	Blood oxygen level measurement.	[20, 100]	0	Num.
14	Respiratory Rate	Breaths per minute measurement.	[10, 99]	0	Num.
15	Heart Rate	Pulse rate measurement per minute.	[20.0, 264.0]	0	Num.
16	Systolic Blood Pressure	Maximum arterial pressure measurement.	[0.0, 237.0]	0	Num.
17	Diastolic Blood Pressure	Minimum arterial pressure measurement.	[0.0, 157.0]	0	Num.
18	Temperature	The body’s measured temperature.	[34.6, 39.5]	0	Num.

**Table 2:** Intervals created in the LOS-DAYS-LOG-CATEGORY feature.

Class Label	Interval	Frequency
0	(0.0, 2.0]	1301
1	(2.0, 6.0]	1392
2	(6.0, 11.0]	1033
3	(11.0, 24.0]	1204

**Table 3:** Description of different feature sets used in the experiments.

Abbreviation	Feature Selection Method	No. Features
FS-1	None	69
FS-2	Pearson	18
FS-3	Kruskal (R)	37
FS-4	Kruskal (C)	24
FS-5	Chi-square	39
FS-6	Pearson & Kruskal (R)	55
FS-7	Kruskal (C) & chi-square	63
FS-8	Kruskal (C) & Kruskal (R)	61
FS-9	Chi-square & Pearson	57
FS-10	Chi-square & Kruskal (R)	76

**Table 4:** Performance comparison of the best-performing models for the patient LOS regression task, showing the $R^{2}$, MSE, MAE, and RMSE scores along with the standard deviation (STD).

Metric		RF	XGBoost	ANN	AvgVotReg	2LSCG	3LSCG	PCG
$R^{2}$	Mean	0.5087	0.4575	0.4594	**0.5185**	0.4895	0.5004	0.5066
	Max	0.5505	0.4915	0.5154	**0.5638**	0.5598	0.5532	0.5432
	STD	0.0247	0.0251	0.0426	0.0282	0.0429	0.0351	0.0233
MSE	Mean	0.0482	0.0532	0.0530	**0.0472**	0.0500	0.0490	0.0483
	Min	0.0447	0.0506	0.0476	**0.0434**	0.0438	0.0444	0.0454
	STD	0.0024	0.0025	0.0045	0.0028	0.0035	0.0033	0.0021
RMSE	Mean	0.2194	0.2306	0.2301	**0.2172**	0.2235	0.2212	0.2199
	Min	0.2115	0.2249	0.2182	**0.2083**	0.2092	0.2108	0.2132
	STD	0.0056	0.0054	0.0098	0.0066	0.0079	0.0075	0.0049
MAE	Mean	0.1728	0.1726	0.1847	0.1703	0.1755	**0.1700**	0.1731
	Min	0.1688	0.1681	0.1705	0.1652	0.1651	**0.1628**	0.1664
	STD	0.0036	0.0040	0.0099	0.0042	0.0057	0.0062	0.0050

## Data Availability

The dataset used in this study is proprietary and not available publicly due to data protection regulations.

## Abbreviations

The following abbreviations are used in this manuscript:

2LSCG	Two-level sequential cascade generalization
3LSCG	Three-level sequential cascade generalization
ANN	Artificial neural network
AvgVotReg	Average voting regression
CNN	Convolutional neural network
DL	Deep learning
FS	Feature subset
ICU	Intensive care unit
IQR	interquartile range
KFSH & RC	King Faisal Specialist Hospital & Research Center
LOS	Length of stay
ML	Machine learning
PCG	Parallel cascade generalization
RF	Random forest
SVM	Support vector machine
XGBoost	Extreme gradient boosting
